# Supplementary material for: The Effect of Proinflammatory Cytokines on the Proliferation, Migration and Secretory Activity of Mesenchymal Stem/Stromal Cells (WJ-MSCs) under 5% O2 and 21% O2 Culture Conditions
Source: J Clin Med. 2021 Apr 21;10(9):1813. doi: 10.3390/jcm10091813 (PMC8122617; doi:10.3390/jcm10091813)
Supplement: Supplementary file 1 [file jcm-10-01813-s001.zip › jcm-1131454-supplementary.pdf]

Journal of
Clinical Medicine

MDPI

Table S1. Primary Antibodies Used in Immunocytochemistry.

Primary antibodies	Isotype	Dilution	Company	Catalogue number
Polyclonal anti- Fibronectin (rabbit)	IgG (H + L)	1:500	Sigma-Aldrich	AB1620
Monoclonal anti- Vimentin (mouse)	IgG1	1:200	Dako	F3648

Table S2. Secondary Antibodies Used in Immunocytochemistry.

Secondary antibodies	Conjugate	Dilution	Company	Catalogue number
Goat anti-rabbit IgG (H + L)	Alexa Fluor 488	1:1000	Invitrogen	R37116
Goat anti-mouse IgG1	Alexa Fluor 488	1:1000	Invitrogen	A-10631

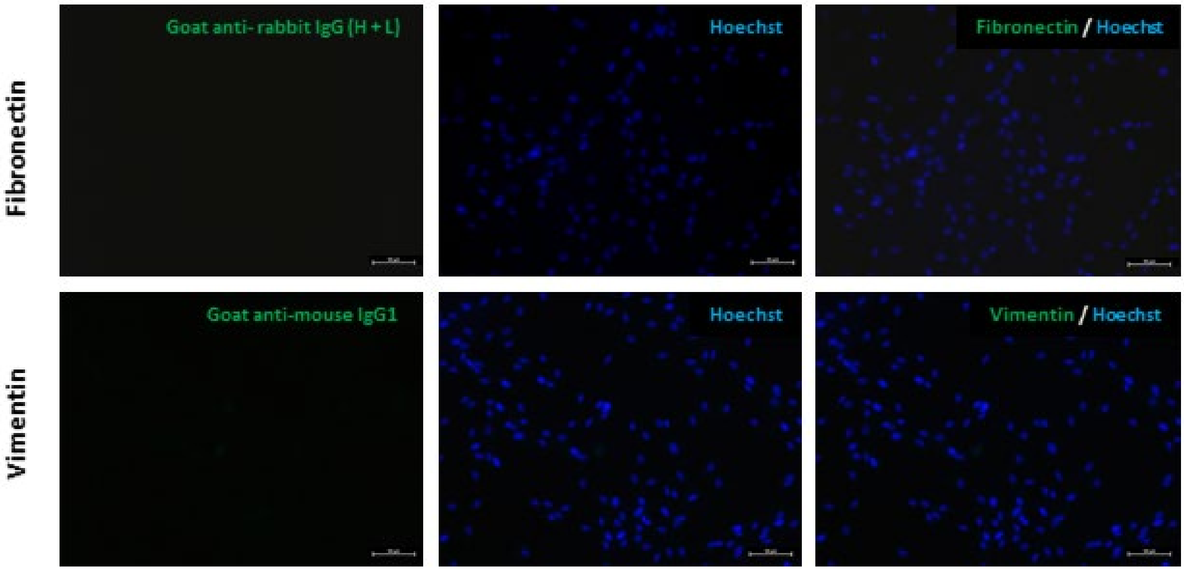

Figure S1. Secondary antibody staining controls. Following secondary antibodies were applies: goat anti-rabbit IgG (H + L) for fibronectin staining and goat anti-mouse IgG1 for vimentin staining. Following conjugate was applied: Alexa Fluor 488—green label for both stainings. Cell nuclei were stained Hoechst 33258. Scale bar: 50 µm.
